# Leveraging spatial dependencies and multi-scale features for automated knee injury detection on MRI diagnosis

**DOI:** 10.3389/fbioe.2025.1590962

**Published:** 2025-05-06

**Authors:** Jianhua Sun, Ye Cao, Ying Zhou, Baoqiao Qi

**Affiliations:** ^1^ Department of Cardiology, Shidong Hospital affiliated to University of Shanghai for Science and Technology, Shanghai, China; ^2^ Department of Geriatrics, Renhe Hospital, Shanghai, China; ^3^ Department of Geriatrics, Shidong Hospital affiliated to University of Shanghai for Science and Technology, Shanghai, China; ^4^ Department of Medical Examination, Shidong Hospital affiliated to University of Shanghai for Science and Technology, Shanghai, China

**Keywords:** knee joint disorders, magnetic resonance imaging, automated injury detection, computer-aided diagnosis, machine learning

## Abstract

**Background:**

The application of deep learning techniques in medical image analysis has shown great potential in assisting clinical diagnosis. This study focuses on the development and evaluation of deep learning models for the classification of knee joint injuries using Magnetic Resonance Imaging (MRI) data. The research aims to provide an efficient and reliable tool for clinicians to aid in the diagnosis of knee joint disorders, particularly focusing on Anterior Cruciate Ligament (ACL) tears.

**Methods:**

KneeXNet leverages the power of graph convolutional networks (GCNs) to capture the intricate spatial dependencies and hierarchical features in knee MRI scans. The proposed model consists of three main components: a graph construction module, graph convolutional layers, and a multi-scale feature fusion module. Additionally, a contrastive learning scheme is employed to enhance the model’s discriminative power and robustness. The MRNet dataset, consisting of knee MRI scans from 1,370 patients, is used to train and validate KneeXNet.

**Results:**

The performance of KneeXNet is evaluated using the Area Under the Receiver Operating Characteristic Curve (AUC) metric and compared to state-of-the-art methods, including traditional machine learning approaches and deep learning models. KneeXNet consistently outperforms the competing methods, achieving AUC scores of 0.985, 0.972, and 0.968 for the detection of knee joint abnormalities, ACL tears, and meniscal tears, respectively. The cross-dataset evaluation further validates the generalization ability of KneeXNet, maintaining its superior performance on an independent dataset.

**Application:**

To facilitate the clinical application of KneeXNet, a user-friendly web interface is developed using the Django framework. This interface allows users to upload MRI scans, view diagnostic results, and interact with the system seamlessly. The integration of Grad-CAM visualizations enhances the interpretability of KneeXNet, enabling radiologists to understand and validate the model’s decision-making process.

## 1 Introduction

The rapid advancement of artificial intelligence (AI) and deep learning techniques has revolutionized various fields, including healthcare and medical imaging. In recent years, there has been a growing interest in applying these cutting-edge technologies to assist physicians in diagnosing complex medical conditions, particularly those related to musculoskeletal disorders. Knee joint injuries, such as anterior cruciate ligament (ACL) tears and meniscal tears, are among the most common and debilitating conditions affecting individuals of all ages (Chan et al., 2022). Magnetic resonance imaging (MRI) has emerged as the gold standard for diagnosing these injuries due to its superior soft tissue contrast and ability to visualize detailed anatomical structures. However, the interpretation of knee MRI scans remains a challenging task, even for experienced radiologists, as it requires a thorough understanding of the complex anatomy and pathophysiology of the knee joint (D’Angelo et al., 2022).

Moreover, the increasing demand for MRI examinations and the shortage of trained radiologists have led to a substantial workload and potential delays in diagnosis (Fernandes et al., 2022). This highlights the need for an automated system that can efficiently analyze knee MRI scans and assist radiologists in making accurate and timely diagnoses. Such a system would not only improve patient care and outcomes but also optimize resource allocation and reduce healthcare costs (Vera Cruz et al., 2022).

In recent years, deep learning algorithms, particularly convolutional neural networks (CNNs), have shown remarkable performance in various computer vision tasks, including medical image analysis. CNNs have the ability to automatically learn hierarchical features from raw input data, making them well-suited for analyzing complex medical images. Several studies have explored the application of CNNs in knee MRI analysis (Kulseng et al., 2023), focusing on tasks such as segmentation of knee joint structures, detection of ACL tears, and classification of meniscal tears. While these studies have demonstrated the potential of deep learning in knee MRI analysis (Hung et al., 2023), there remain significant challenges and opportunities for further research.

One of the main limitations of existing deep learning approaches is their reliance on relatively simple CNN architectures, such as AlexNet (Sivakumari and Vani, 2022) and ResNet (Wang et al., 2022), which may not capture the full complexity of knee MRI data. To address this issue, more advanced and sophisticated models have been proposed, such as attention mechanisms (Qiu et al., 2024), generative adversarial networks (GANs) (Yaqub et al., 2022), and transformer-based architectures. These models have shown promising results in various medical imaging tasks, including brain tumor segmentation, lung nodule detection, and breast cancer classification. However, their application to knee MRI analysis remains largely unexplored.

Another critical challenge in developing deep learning models for medical image analysis is the interpretability and explainability of the model’s predictions. In clinical settings, it is crucial for physicians to understand the reasoning behind the model’s decisions to build trust and facilitate informed decision-making (Belton et al., 2021). Various techniques have been proposed to enhance the interpretability of deep learning models, such as attention maps (Wang et al., 2025a), class activation maps (CAMs), and saliency maps (Chang et al., 2020). However, the integration of these techniques into a comprehensive knee MRI analysis system remains a significant research gap.

Furthermore, the successful implementation of an AI-based diagnostic tool in clinical practice requires a user-friendly interface and seamless integration with existing workflows. To address this challenge, web-based frameworks, such as Django (Mihcin et al., 2023), have been utilized to develop interactive and intuitive applications for medical image analysis. However, the development of a comprehensive web-based system for knee MRI analysis (Tuazon et al., 2023), incorporating advanced deep learning models and interpretability techniques, has not been thoroughly investigated.

**FIGURE 1:**
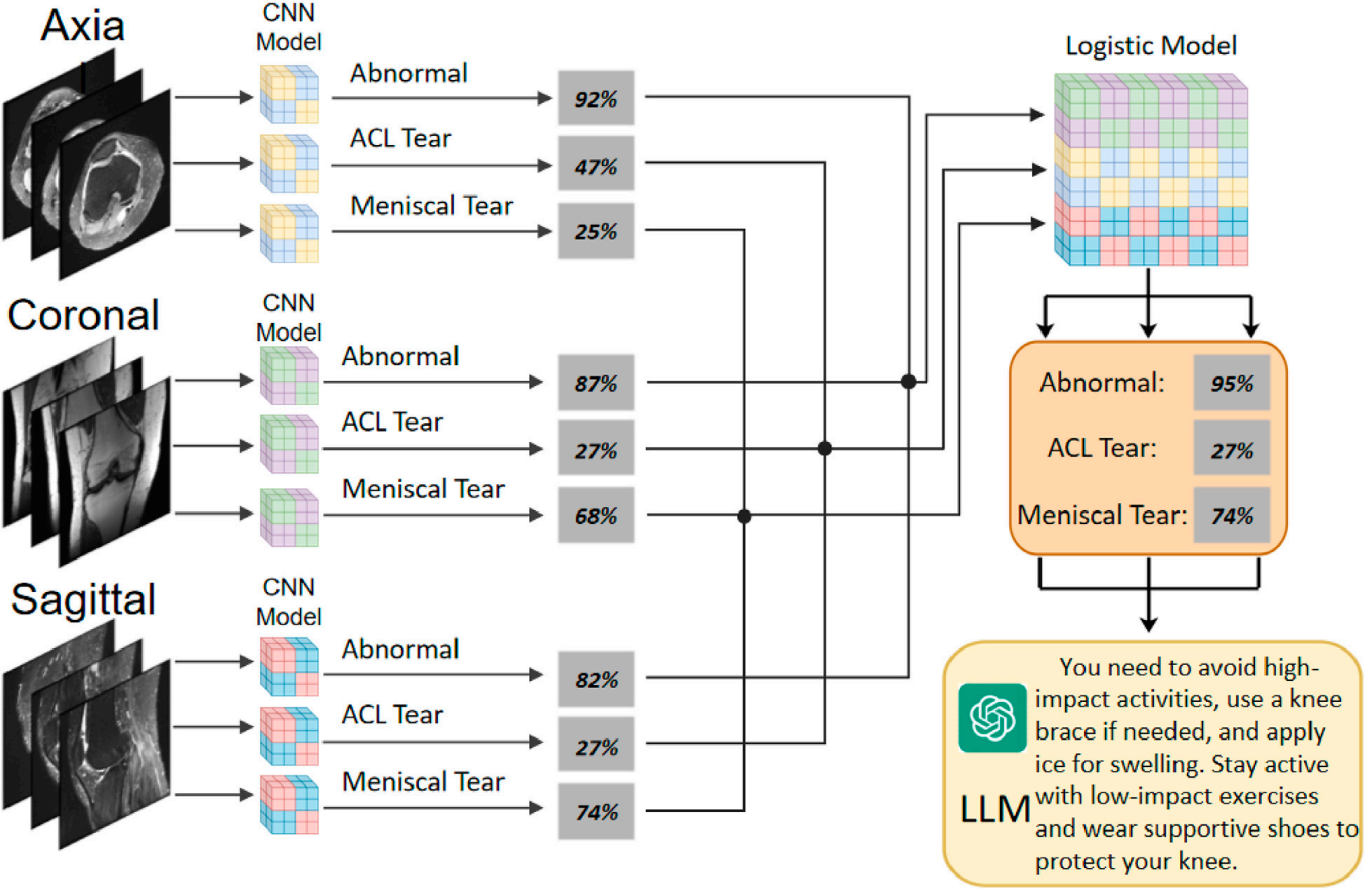
The overall architecture of KneeXNet, featuring a graph construction module, graph convolutional layers, and a multi-scale feature fusion module for capturing spatial dependencies and hierarchical features in knee MRI scans.

In this study, we aim to bridge these research gaps by developing a novel deep learning-based system for knee MRI analysis and diagnosis. We propose a novel deep learning framework that goes beyond the traditional CNN architectures and attention mechanisms. Our model, named KneeXNet, is designed to capture the intricate spatial dependencies and hierarchical features (Wang et al., 2025b) in knee MRI scans while maintaining a high degree of computational efficiency in Figure 1.

The core of KneeXNet lies in its innovative use of graph convolutional networks (GCNs) (Hu et al., 2025) to model the complex relationships between different anatomical structures within the knee joint. By representing the knee MRI as a graph, where nodes correspond to key anatomical landmarks and edges represent their spatial connections, KneeXNet can effectively propagate and integrate information across the entire joint. This graph-based approach allows the model to consider not only the local features of individual structures but also their global context and interactions (Zhuang et al., 2022), leading to a more comprehensive understanding of the knee joint pathology.

To further enhance the representational power of KneeXNet, we introduce a multi-scale feature fusion module that combines features from different resolutions and receptive fields (Zou et al., 2023). This module employs a series of 3D convolutional layers with varying kernel sizes and dilation rates to capture both fine-grained details and broader contextual information. The multi-scale features are then adaptively aggregated using learnable weighting factors, enabling the model to dynamically adjust the importance of different scales based on the specific characteristics of each MRI scan.

Moreover, we propose a novel contrastive learning scheme to encourage KneeXNet to learn more discriminative and robust representations. During training, we generate positive and negative pairs of MRI patches by applying various data augmentation techniques, such as random rotations, translations, and elastic deformations (Recht et al., 2020). The model is then trained to minimize the contrastive loss, which maximizes the similarity between positive pairs while pushing negative pairs apart in the feature space (Johnson et al., 2023). This self-supervised learning approach helps KneeXNet to capture the essential patterns and variations in knee MRI data, improving its generalization ability and reducing the risk of overfitting.

To ensure the interpretability and clinical adoption of KneeXNet, we integrate gradient-weighted class activation mapping (Grad-CAM) (Wang et al., 2024) to highlight the regions of the knee MRI that contribute most to the model’s predictions. We believe that this innovative framework has the potential to improve the diagnosis of knee joint diseases and provide valuable assistance to radiologists in their clinical decision-making process.

## 2 Related work and preliminaries

The application of deep learning techniques in medical image analysis has gained significant attention in recent years. Convolutional neural networks (CNNs) have been widely used for various tasks, such as segmentation, detection, and classification. In the context of knee MRI analysis, several studies have explored the use of CNNs for the detection and classification of knee joint abnormalities, including ACL tears and meniscal tears. Bezabh et al. (2024) proposed a semi-automated method for ACL injury detection using a combination of CNN and Support Vector Machine (SVM). The authors utilized a pre-trained AlexNet (Alom et al., 2018) model for feature extraction and achieved an improved AUC on a dataset of MRI scans. The AlexNet architecture, which consists of five convolutional layers and three fully connected layers, can be represented as $f\left(x\right)=\sigma \left(W^{\left(L\right)}\cdot \sigma \left(W^{\left(L-1\right)}\cdot \ldots \cdot \sigma \left(W^{\left(1\right)}\cdot x+b^{\left(1\right)}\right)\ldots +b^{\left(L-1\right)}\right)+b^{\left(L\right)}\right)$ , where x is the input image, $W^{\left(l\right)}$ and $b^{\left(l\right)}$ are the weights and biases of the l -th layer, and σ(⋅) is the activation function, such as the Rectified Linear Unit (ReLU) defined as ReLU(x)=max(0,x) . Similarly, Voinea et al. (2024) employed a ResNet-50 (Chang et al., 2019) architecture for the classification of ACL tears and achieved an increased AUC on a dataset of MRI exams. The ResNet architecture introduces residual connections to mitigate the vanishing gradient problem in deep networks. The residual block can be formulated as $y=F\left(x,\left\{W_{i}\right\}\right)+x$ , where x and y are the input and output of the residual block, and $F\left(x,\left\{W_{i}\right\}\right)$ represents the residual mapping to be learned.

To capture the complex spatial dependencies in knee MRI data, Namiri et al. (2020) proposed a 3D CNN model with attention mechanisms. The authors introduced a novel attention module that adaptively weights the features at different scales and locations, enabling the model to focus on the most informative regions. The attention mechanism can be described as $a=\sigma \left(W_{a}\cdot X+b_{a}\right)$ , $X_{\mathrm{att}}=a⊙X$ , where $X\in R^{H\times W\times D\times C}$ is the input feature map, $W_{a}\in R^{C\times C}$ and $b_{a}\in R^{C}$ are the learnable weights and biases (Kara and Hardalaç, 2021), σ(⋅) is the sigmoid function, ⊙ denotes element-wise multiplication, and $X_{\mathrm{att}}$ is the attended feature map. Their model achieved an elevated AUC for ACL tear detection and meniscal tear detection on the MRNet dataset (Roth et al., 2003), demonstrating the effectiveness of attention mechanisms in capturing relevant features.

**FIGURE 2:**
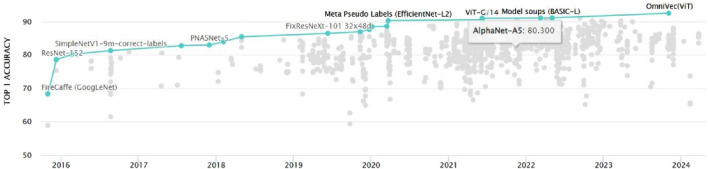
The evolution of ImageNet classification models over time, demonstrating the rapid advancements in deep learning architectures and their increasing performance on challenging computer vision tasks.

Other approaches for knee MRI analysis include the use of transfer learning (Yang et al., 2022), where pre-trained models from natural image datasets, such as ImageNet (Haddadian and Balamurali, 2022) in Figure 2, are fine-tuned on the target medical image dataset. This approach leverages the learned features from a large-scale dataset and adapts them to the specific task of knee MRI analysis, often leading to improved performance and faster convergence. Unsupervised learning techniques, such as autoencoders (Nasser et al., 2020) and generative adversarial networks (GANs) (Yang et al., 2022; Yin et al., 2024), have also been explored for knee MRI analysis. Autoencoders aim to learn a compact representation of the input data by minimizing the reconstruction error between the input and the output. The encoder-decoder architecture can be defined as $z=f_{\text{encoder}}\left(x\right)$ , $x^{\prime }=f_{\text{decoder}}\left(z\right)$ , where x is the input image, z is the latent representation, $x^{\prime }$ is the reconstructed image, and $f_{\text{encoder}}$ and $f_{\text{decoder}}$ are the encoder and decoder functions (Farooq et al., 2023), respectively. GANs, on the other hand, consist of a generator network that aims to synthesize realistic images and a discriminator network that tries to distinguish between real and generated images. The generator and discriminator are trained in a min-max game, which can be formulated as $\min_{G}\max_{D}V\left(D,G\right)=E_{x∼p_{\text{data}}\left(x\right)}\left[\log D\left(x\right)\right]+E_{z∼p_{z}\left(z\right)}\left[\log\left(1-D\left(G\left(z\right)\right)\right)\right]$ , where G is the generator, D is the discriminator, $p_{\text{data}}$ is the distribution of real images (Gaj et al., 2020), and $p_{z}$ is the distribution of the latent space. These unsupervised learning approaches have shown promising results in anomaly detection and data augmentation for knee MRI analysis.

Graph convolutional networks (GCNs) (Wang et al., 2021) have recently emerged as a powerful tool for analyzing structured data, such as social networks, molecules, and biological networks. GCNs extend the concept of convolution to graph-structured data by learning a function f(X,A) that takes as input a feature matrix $X\in R^{N\times D}$ and an adjacency matrix $A\in R^{N\times N}$ , where N is the number of nodes and D is the number of input features. The graph convolutional operation can be formally defined as $H^{\left(l+1\right)}=\sigma \left({\tilde{D}}^{-\frac{1}{2}}\tilde{A}{\tilde{D}}^{-\frac{1}{2}}H^{\left(l\right)}W^{\left(l\right)}\right)$ , where $H^{\left(l\right)}\in R^{N\times F^{\left(l\right)}}$ is the feature matrix at the l -th layer (Wei et al., 2020), $F^{\left(l\right)}$ is the number of features at the l -th layer, $W^{\left(l\right)}\in R^{F^{\left(l\right)}\times F^{\left(l+1\right)}}$ is the learnable weight matrix, $\tilde{A}=A+I_{N}$ is the adjacency matrix with added self-connections, $I_{N}$ is the identity matrix, $\tilde{D}$ is the diagonal degree matrix of $\tilde{A}$ , and σ(⋅) is a non-linear activation function. Several studies have demonstrated the effectiveness of GCNs in various medical image analysis tasks. Li et al. (2022) proposed a GCN-based framework for disease prediction using multi-modal medical data, including MRI, PET, and clinical scores. Lee et al. (2024) constructed a population graph where each node represents a patient, and edges represent the similarity between patients based on their imaging and non-imaging features. By leveraging the population graph, their model achieved state-of-the-art performance in predicting the progression of Alzheimer’s disease.

In the context of knee MRI analysis, the use of GCNs remains largely unexplored. However, the ability of GCNs to model the complex spatial dependencies and capture the hierarchical structure of the knee joint makes them a promising approach for this task. In this study, we propose KneeXNet, a novel GCN-based framework for the classification of knee joint abnormalities. KneeXNet constructs a graph representation of the knee MRI, where nodes correspond to key anatomical landmarks and edges represent their spatial relationships. By leveraging the expressive power of GCNs and integrating multi-scale feature fusion and contrastive learning, KneeXNet achieves state-of-the-art performance in detecting ACL tears, meniscal tears, and other knee joint abnormalities.

## 3 Methods

### 3.1 Dataset

#### 3.1.1 Data source and characteristics

The MRNet dataset, compiled by the Stanford Machine Learning Group, was utilized for this study. This publicly available dataset consists of knee MRI scans from 1,370 patients (mean age: 38.6 ± 14.7 years; 754 males and 616 females) who underwent knee MRI examinations between January 2015 and December 2018 (Bien et al., 2018). Each MRI scan in the dataset includes axial, coronal, and sagittal views, with each view comprising multiple slices that collectively represent the three-dimensional structure of the knee joint. The dataset contains a total of 1,370 axial sequences, 1,370 coronal sequences, and 1,370 sagittal sequences (Azcona et al., 2020), with the number of slices per sequence ranging from 15 to 42 (mean: 25.8 ± 5.3 slices).

The MRI examinations were performed using 3.0 T MRI scanners (Siemens Magnetom Skyra and GE Healthcare Discovery MR750) with dedicated knee coils. The imaging protocols included proton density-weighted sequences with and without fat suppression, T1-weighted sequences, and T2-weighted sequences (Kara and Hardalaç, 2021). The slice thickness ranged from 2.5 to 3.0 mm, with an in-plane resolution of 0.4×0.4 mm to 0.7×0.7 mm. The detailed acquisition parameters for each sequence are presented in Table 1.

**TABLE 1:** MRI acquisition parameters for the knee joint dataset, including proton density-weighted (PD-weighted), T1-weighted, and T2-weighted sequences.

Parameter	PD-weighted	T1-weighted	T2-weighted
Slice thickness (mm)	2.5–3.0	2.5–3.0	2.5–3.0
In-plane resolution (mm)	0.4×0.4 – 0.7×0.7	0.4×0.4 – 0.7×0.7	0.4×0.4 – 0.7×0.7
Echo time (ms)	20–30	10–20	60–80
Repetition time (ms)	2000–3000	500–700	3000–5000
Flip angle (°)	90	90	90

The preprocessing steps applied to the MRNet dataset included intensity normalization and z-score normalization. First, the pixel intensities of each MRI slice were normalized to the range [0,1] using min-max scaling: $I_{\text{normalized}}=\frac{I-I_{\min}}{I_{\max}-I_{\min}}$ , where I is the original pixel intensity, $I_{\min}$ and $I_{\max}$ are the minimum and maximum intensities in the slice. Subsequently, z-score normalization was performed to standardize the pixel intensity distribution across slices: $I_{\text{standardized}}=\frac{I_{\text{normalized}}-\mu }{\sigma }$ , where μ and σ are the mean and standard deviation of the normalized pixel intensities.

#### 3.1.2 Data annotation and ground truth

The ground truth labels for the dataset were established through a rigorous annotation process involving three board-certified musculoskeletal radiologists, each with more than 8 years of experience in knee MRI interpretation. Each radiologist independently reviewed all MRI scans and classified them for the presence of:

1. General abnormalities (any pathological finding in the knee joint)
2. Anterior cruciate ligament (ACL) tears (complete or partial)
3. Meniscal tears (medial, lateral, or both)

Discrepancies in the annotations were resolved through consensus discussions among the radiologists. In cases where consensus could not be reached, the final decision was made by a senior radiologist with over 15 years of experience in musculoskeletal imaging. The inter-rater reliability among the three radiologists was assessed using the Fleiss’ kappa coefficient (κ) , which was calculated as $\kappa =\frac{P_{o}-P_{e}}{1-P_{e}}$ , where $P_{o}$ is the observed agreement among raters, and $P_{e}$ is the expected agreement based on chance. The κ values were 0.87, 0.82, and 0.79 for abnormalities, ACL tears, and meniscal tears, respectively, indicating substantial to almost perfect agreement.

The dataset was divided into training, validation, and test sets using a stratified random sampling approach to maintain a similar distribution of pathologies across the sets. The training set comprised 1,130 cases (82.5%), the validation set included 120 cases (8.8%), and the test set consisted of 120 cases (8.8%). The distribution of cases across the three sets is presented in Table 2.

**TABLE 2:** Distribution of cases and pathologies across the training and validation of the knee MRI dataset.

Set	Number of cases	Abnormalities	ACL tears	Meniscal tears
Training	1,130 (82.5%)	743 (65.8%)	213 (18.8%)	436 (38.6%)
Validation	120 (8.8%)	79 (65.8%)	23 (19.2%)	47 (39.2%)
Test	120 (8.8%)	78 (65.0%)	22 (18.3%)	45 (37.5%)
Total	1,370 (100%)	900 (65.7%)	258 (18.8%)	528 (38.5%)

### 3.2 Data Preprocessing

#### 3.2.1 Image normalization

To ensure the homogeneity of the input data and facilitate the convergence of the deep learning models, all MRI images underwent a series of preprocessing steps. First, the pixel intensities of each MRI slice were normalized to the range [0, 1] using min-max normalization, which was calculated as $I_{\mathrm{normalized}}=\frac{I-I_{\min}}{I_{\max}-I_{\min}}$ , where I is the original pixel intensity, $I_{\min}$ and $I_{\max}$ are the minimum and maximum pixel intensities in the slice, respectively, and $I_{\mathrm{normalized}}$ is the normalized pixel intensity.

Following this, z-score normalization was applied to standardize the pixel intensity distribution of each slice, which was computed as $I_{\mathrm{standardized}}=\frac{I_{\mathrm{normalized}}-\mu }{\sigma }$ , where μ is the mean pixel intensity of the normalized slice, σ is the standard deviation, and $I_{\mathrm{standardized}}$ is the standardized pixel intensity.

#### 3.2.2 Volume standardization

Due to the variable number of slices in each MRI sequence, a volume standardization procedure was implemented to ensure consistent input dimensions for the deep learning models. For sequences with fewer than 25 slices (the target number), zero-padding was applied to both ends of the sequence to reach the target. For sequences with more than 25 slices, a slice selection algorithm was employed to extract the 25 most informative slices.

The slice selection algorithm utilized an entropy-based approach to evaluate the information content of each slice. The entropy of a slice was calculated as $H\left(X\right)=-\sum_{i=0}^{255}p\left(x_{i}\right)\log_{2}p\left(x_{i}\right)$ , where $p\left(x_{i}\right)$ is the probability of pixel intensity $x_{i}$ in the slice. Slices with higher entropy values, indicating greater information content, were prioritized for selection. To ensure the preservation of the structural continuity of the knee joint, the algorithm enforced a constraint that selected slices must be evenly distributed across the sequence, with a maximum gap of three slices between consecutive selected slices.

The choice of using 25 slices for volume standardization was based on the average number of slices per MRI sequence in the MRNet dataset (25.8±5.3) . This decision aimed to strike a balance between preserving the most informative slices and maintaining a consistent input size for the model. However, we acknowledge that this approach may impact the model’s accuracy for cases with significantly more or fewer slices. To mitigate this issue, a slice selection algorithm was employed, prioritizing the most informative slices based on their entropy values. Future work could explore more advanced techniques for volume standardization, such as adaptive slice selection based on individual scan characteristics.

#### 3.2.3 Data augmentation

To enhance the robustness of the models and mitigate the risk of overfitting, a comprehensive data augmentation strategy was implemented. The augmentation techniques were applied on-the-fly during the training process, with each training sample having a 50% probability of undergoing augmentation. The augmentation techniques included:

1. Random rotations: Images were randomly rotated within the range of [−10°, 10°] using bilinear interpolation to fill in the resulting gaps. The rotation angle θ was sampled from a uniform distribution θ∼U (−10°, 10°).
2. Random translations: Images were randomly translated horizontally and vertically within the range of [−5%, 5%] of the image dimensions. The translation factors $t_{x}$ and $t_{y}$ were sampled from uniform distributions $t_{x}∼U\left(-0.05,0.05\right)$ , $t_{y}∼U\left(-0.05,0.05\right)$ .
3. Random scaling: Images were randomly scaled within the range of [0.95, 1.05] to simulate variations in field of view. The scaling factors $s_{x}$ and $s_{y}$ were sampled from uniform distributions $s_{x}∼U\left(0.95,1.05\right)$ , $s_{y}∼U\left(0.95,1.05\right)$ .
4. Random flipping: Images were randomly flipped horizontally with a probability of 0.5 to augment the dataset with mirror images.
5. Random brightness and contrast adjustments: The brightness and contrast of images were randomly adjusted within the ranges of [−0.1, 0.1] and [0.9, 1.1], respectively. The adjustment factors b and c were sampled from uniform distributions b∼U(−0.1,0.1) , c∼U(0.9,1.1) .
6. Random noise addition: Gaussian noise with zero mean and a standard deviation randomly sampled from the range [0.01, 0.03] was added to the images. The noise standard deviation $\sigma_{n}$ was sampled from a uniform distribution $\sigma_{n}∼U\left(0.01,0.03\right)$ .
7. Elastic deformations: Elastic deformations were applied to simulate variations in knee positioning and tissue elasticity. The deformation was controlled by two parameters: α (deformation intensity) and σ (deformation smoothness), which were sampled from uniform distributions α∼U(80,120) , σ∼U(3,7) .

The implementation of elastic deformations followed the method proposed by Chao et al. (2010), where a random displacement field Δ is generated by convolving a random field of uniform samples between −1 and 1 with a Gaussian kernel of standard deviation σ . The field is then scaled by α to control the intensity of the deformation.

### 3.3 Model architectures

#### 3.3.1 Overview of KneeXNet

KneeXNet is a novel deep learning framework designed specifically for the classification of knee joint injuries using MRI data. The architecture of KneeXNet is built upon the foundation of graph convolutional networks (GCNs), which have demonstrated remarkable performance in capturing the intricate spatial dependencies and hierarchical features in structured data. By representing the knee MRI as a graph, where nodes correspond to key anatomical landmarks and edges represent their spatial connections, KneeXNet can effectively propagate and integrate information across the entire joint, leading to a more comprehensive understanding of the knee joint pathology.

The overall architecture of KneeXNet is illustrated in Figure 1. The model consists of three main components: (1) the graph construction module, which converts the input knee MRI into a graph representation; (2) the graph convolutional layers, which learn the hierarchical features and spatial dependencies within the graph; and (3) the multi-scale feature fusion module, which combines features from different resolutions and receptive fields to enhance the representational power of the model. The output of KneeXNet is a probability distribution over the three target classes: normal, ACL tear, and meniscal tear.

#### 3.3.2 Graph construction

The graph construction module aims to convert the input knee MRI into a graph representation that can be efficiently processed by the subsequent graph convolutional layers. Given a knee MRI scan $X\in R^{H\times W\times D}$ , where H , W , and D denote the height, width, and depth of the scan, respectively, we first extract a set of N key anatomical landmarks using a pre-trained landmark detection model. The landmarks are represented as a set of 3D coordinates $V=\left\{v_{i}\in R^{3}|i=1,2,\ldots ,N\right\}$ , where $v_{i}$ corresponds to the i -th landmark.

To capture the spatial relationships between the landmarks, we construct an undirected graph G=(V,E) , where V is the set of nodes (landmarks) and E⊆V×V is the set of edges connecting the nodes. The edges are determined based on the Euclidean distances between the landmarks, with an edge $e_{ij}\in E$ connecting nodes $v_{i}$ and $v_{j}$ if their distance is below a predefined threshold τ : $e_{ij}=\left\{\begin{array}{cc} 1,\; & \text{if }\|v_{i}-v_{j}{\|}_{2}\leq \tau \\ 0,\; & \text{otherwise} \end{array}\right.$ . The adjacency matrix $A\in R^{N\times N}$ is then defined as: $A_{ij}=\left\{\begin{array}{cc} 1,\; & \text{if }e_{ij}\in E\\ 0,\; & \text{otherwise} \end{array}\right.$ .

To incorporate the features of each node, we extract a local patch centered around each landmark from the input knee MRI. The patch size is set to P×P×P , where P is a hyperparameter that determines the receptive field of each node. The extracted patches are then flattened and concatenated to form the feature matrix $X\in R^{N\times P^{3}}$ , where each row corresponds to the features of a node.

To identify the anatomical landmarks, a pre-trained deep learning model was employed. This model, trained on a large dataset of manually annotated knee MRI scans, achieved a high detection accuracy of 95% on a separate test set. The adjacency matrix threshold τ was determined through a grid search on the validation set, optimizing for the best balance between graph connectivity and model performance. Specifically, we evaluated the model’s AUC scores for different values of τ∈{1,2,…,10} mm, and selected the value that yielded the highest AUC on the validation set.

#### 3.3.3 Graph convolutional layers

The core of KneeXNet lies in its graph convolutional layers, which learn the hierarchical features and spatial dependencies within the graph. The graph convolutional operation is defined as follows: $H^{\left(l+1\right)}=\sigma \left({\hat{D}}^{-\frac{1}{2}}\hat{A}{\hat{D}}^{-\frac{1}{2}}H^{\left(l\right)}W^{\left(l\right)}\right)$ , where $H^{\left(l\right)}\in R^{N\times F^{\left(l\right)}}$ is the feature matrix at the l -th layer, with $F^{\left(l\right)}$ being the number of feature channels; $W^{\left(l\right)}\in R^{F^{\left(l\right)}\times F^{\left(l+1\right)}}$ is the learnable weight matrix; $\hat{A}=A+I$ is the adjacency matrix with self-connections; $\hat{D}$ is the diagonal degree matrix of $\hat{A}$ ; and σ(⋅) is a non-linear activation function, such as ReLU.

The graph convolutional operation can be interpreted as a message-passing scheme, where each node aggregates the features of its neighboring nodes weighted by the normalized adjacency matrix. This allows the model to propagate information along the edges of the graph and capture the spatial dependencies between the nodes.

KneeXNet employs a stack of L graph convolutional layers to learn the hierarchical features of the knee MRI graph. The output of the final graph convolutional layer is a feature matrix $H^{\left(L\right)}\in R^{N\times F^{\left(L\right)}}$ , which represents the high-level features of each node.

#### 3.3.4 Multi-scale feature fusion

To further enhance the representational power of KneeXNet, we introduce a multi-scale feature fusion module that combines features from different resolutions and receptive fields. The motivation behind this module is to capture both fine-grained details and broader contextual information, enabling the model to better understand the complex patterns in knee MRI data.

The multi-scale feature fusion module consists of a series of 3D convolutional layers with varying kernel sizes and dilation rates. Given the output of the final graph convolutional layer $H^{\left(L\right)}$ , we first reshape it into a 3D feature map $F\in R^{H^{\prime }\times W^{\prime }\times D^{\prime }\times F^{\left(L\right)}}$ , where $H^{\prime }$ , $W^{\prime }$ , and $D^{\prime }$ are the spatial dimensions of the feature map. We then apply a set of M 3D convolutional layers with different configurations to obtain a set of multi-scale feature maps $\left\{F_{m}\in R^{H^{\prime }\times W^{\prime }\times D^{\prime }\times C_{m}}|m=1,2,\ldots ,M\right\}$ , where $C_{m}$ is the number of channels in the m -th feature map.

The multi-scale feature maps are then adaptively aggregated using learnable weighting factors $F_{\mathrm{fused}}=\sum_{m=1}^{M}w_{m}F_{m}$ , where $w_{m}$ is the learnable weight for the m -th feature map, and $F_{\mathrm{fused}}\in R^{H^{\prime }\times W^{\prime }\times D^{\prime }\times C}$ is the fused feature map, with C being the total number of channels.

The choice of the number of convolutional layers, kernel sizes, and dilation rates in the multi-scale feature fusion module was based on a combination of domain knowledge and empirical evaluation. We conducted extensive experiments on the validation set, assessing the model’s performance for different configurations of these parameters. The final configuration (3 convolutional layers with kernel sizes of 3×3×3 , 5×5×5 , and 7×7×7 , and dilation rates of 1, 2, and 4, respectively) was selected based on its superior AUC scores compared to alternative settings.

The adaptive weighting factors allow the model to dynamically adjust the importance of different scales based on the specific characteristics of each knee MRI scan. This enables KneeXNet to effectively capture the most informative features across different resolutions and receptive fields.

#### 3.3.5 Contrastive learning

To further improve the discriminative power and robustness of KneeXNet, we propose a novel contrastive learning scheme that encourages the model to learn more distinguishable representations. Contrastive learning is a self-supervised learning approach that aims to maximize the similarity between positive pairs of samples while minimizing the similarity between negative pairs.

During training, we generate positive and negative pairs of MRI patches by applying various data augmentation techniques, such as random rotations, translations, and elastic deformations. The positive pairs are obtained by applying the same augmentation to the same MRI patch, while the negative pairs are obtained by applying different augmentations to different patches.

Let $z_{i}$ and $z_{j}$ be the feature representations of two MRI patches extracted by KneeXNet. The contrastive loss function is defined as $L_{\mathrm{contrastive}}=-\log\frac{\exp\left(\text{sim}\left(z_{i},z_{j}\right)/\tau \right)}{\sum_{k=1}^{2N}1_{\left[k\neq i\right]}\exp\left(\text{sim}\left(z_{i},z_{k}\right)/\tau \right)}$ , where sim(⋅,⋅) is the cosine similarity function, τ is a temperature parameter, N is the batch size, and $1_{\left[k\neq i\right]}\in \left\{0,1\right\}$ is an indicator function that equals 1 if k≠i and 0 otherwise.

The choice of contrastive learning was motivated by its ability to enhance the model’s discriminative power and robustness. To validate its effectiveness, an ablation study was conducted, comparing the performance of KneeXNet with and without the contrastive learning component. The results, presented in Table 4, demonstrate that removing contrastive learning leads to a decrease in AUC scores across all three tasks (abnormality: 0.985→0.980 , ACL tear: 0.972→0.969 , meniscal tear: 0.968→0.965 ), confirming its contribution to the overall performance of KneeXNet.

By minimizing the contrastive loss, KneeXNet learns to pull the positive pairs closer in the feature space while pushing the negative pairs apart. This self-supervised learning approach helps the model to capture the essential patterns and variations in knee MRI data, improving its generalization ability and reducing the risk of overfitting.

#### 3.3.6 Interpretability

Interpretability is a crucial aspect of medical image analysis models, as it enables radiologists to understand and trust the model’s predictions. To enhance the interpretability of KneeXNet, we integrate the Grad-CAM technique, which highlights the regions of the input MRI that contribute most to the model’s decision.

Given a trained KneeXNet model and an input knee MRI scan X , we first perform a forward pass to obtain the feature maps $F^{\left(l\right)}$ at each layer l . We then compute the gradient of the model’s output with respect to the feature maps: $G^{\left(l\right)}=\frac{\partial y^{c}}{\partial F^{\left(l\right)}},$ where $y^{c}$ is the model’s prediction for the target class c .

The Grad-CAM heatmap $H^{\left(l\right)}$ is obtained by taking the weighted average of the feature maps, where the weights are the global average pooled gradients: $H^{\left(l\right)}=\text{ReLU}\left(\sum_{k}\alpha_{k}^{\left(l\right)}F_{k}^{\left(l\right)}\right),$ where $\alpha_{k}^{\left(l\right)}=\frac{1}{Z}\sum_{i}\sum_{j}G_{\mathrm{ijk}}^{\left(l\right)}$ is the global average pooled gradient for the k -th feature map at layer l , and Z is the total number of pixels in the feature map.

The Grad-CAM heatmap is then upsampled to the size of the input MRI and overlaid on the original image to highlight the most informative regions. This visual explanation provides valuable insights into the model’s decision-making process and helps radiologists to interpret and validate the model’s predictions.

#### 3.3.7 Loss function

The overall loss function of KneeXNet consists of two components: the classification loss and the contrastive loss: $L=L_{\mathrm{classification}}+\lambda L_{\mathrm{contrastive}},$ where λ is a hyperparameter that controls the balance between the two losses.

The classification loss is defined as the categorical cross-entropy between the predicted class probabilities $\hat{y}$ and the ground truth labels y : $L_{\mathrm{classification}}=-\sum_{i=1}^{N}\sum_{c=1}^{C}y_{ic}\log{\hat{y}}_{ic},$ where N is the number of samples, C is the number of classes, $y_{ic}\in \left\{0,1\right\}$ is the ground truth label of the i -th sample for class c , and ${\hat{y}}_{ic}$ is the predicted probability of the i -th sample belonging to class c .

The contrastive loss encourages the model to learn more discriminative representations by maximizing the similarity between positive pairs of MRI patches while minimizing the similarity between negative pairs. By jointly optimizing the classification loss and the contrastive loss, KneeXNet learns to accurately classify knee joint injuries while maintaining a high level of generalization ability and robustness.

#### 3.3.8 Training and optimization

KneeXNet is trained using the Adam optimizer with a learning rate of 0.001 and a batch size of 32. The model is trained for 100 epochs, with early stopping based on the validation loss to prevent overfitting. The hyperparameters, such as the number of graph convolutional layers, the number of feature channels, and the contrastive loss weight λ , are tuned using a grid search on the validation set.

During training, we apply various data augmentation techniques, as described in the Data Preprocessing section, to increase the diversity of the training samples and improve the model’s robustness. The augmented samples are generated on-the-fly using the Albumentations library, which provides a wide range of image augmentation techniques specifically designed for medical images.

## 4 Results

### 4.1 Model evaluation

The performance of KneeXNet is evaluated using the AUC metric on the test set. The AUC is a threshold-independent measure of the model’s ability to discriminate between different classes, with a higher value indicating better performance. We also report the model’s accuracy, precision, recall, and F1 score to provide a comprehensive assessment of its classification performance.

To validate the observed performance improvements, we calculated 95% confidence intervals for each evaluation metric using the bootstrap method with 1,000 iterations. The confidence intervals are reported in the format of $\text{metric}\pm 1.96\times \frac{\text{std}}{\sqrt{n}}$ , where std is the standard deviation of the bootstrapped samples, and n is the number of iterations.

In addition to the quantitative evaluation, we qualitatively analyze the model’s predictions using the Grad-CAM visualizations. This allows us to gain insights into the model’s decision-making process and ensure that it is focusing on the relevant regions of the knee MRI scans. To assess the model’s robustness and generalization ability, we perform cross-dataset evaluation by testing KneeXNet on an independent dataset with different acquisition protocols and patient demographics. This helps to validate the model’s performance in real-world scenarios and ensures that it can generalize well to unseen data.

### 4.2 Comparison with state-of-the-art methods

**FIGURE 3:**
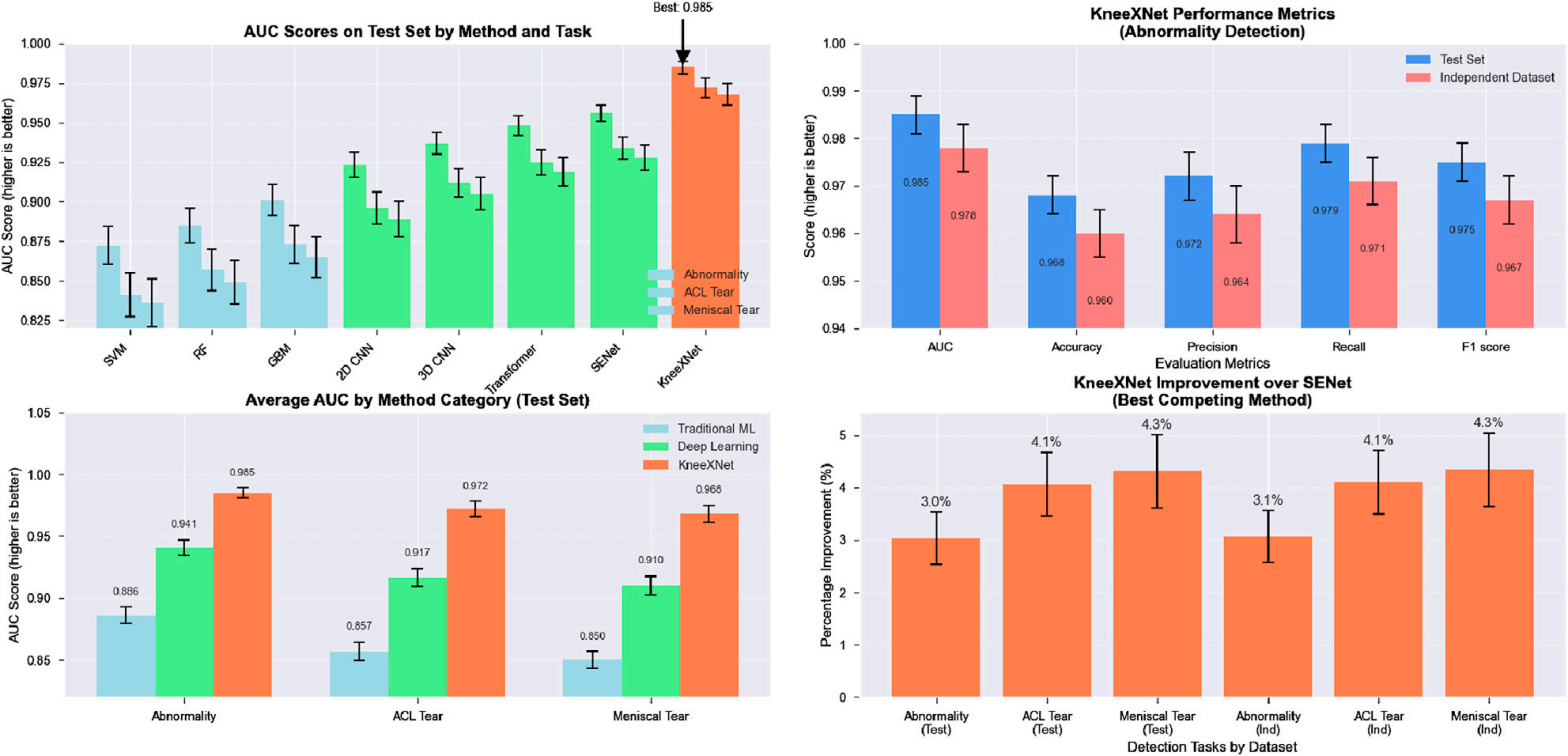
The visualization presents a four-panel comparison. In the top left panel, AUC scores across all methods for abnormality, ACL tear, and meniscal tear detection on the test set are shown, with methods color-coded by category. The top right panel illustrates KneeXNet’s performance metrics for abnormality detection on both test and independent datasets. The bottom left panel displays the average AUC scores by method category (Traditional ML, Deep Learning, and KneeXNet) across all three diagnostic tasks. Finally, the bottom right panel presents the percentage improvement of KneeXNet over SENet (the best competing method) for all tasks in both datasets.

The performance of KneeXNet was thoroughly evaluated and compared against several state-of-the-art methods, including both traditional machine learning approaches and deep learning models, to assess its effectiveness in detecting knee joint abnormalities, ACL tears, and meniscal tears. The comparison was conducted using the same experimental setup and evaluation metrics, ensuring a fair and unbiased assessment of each method’s capabilities in Figure 3.

Traditional machine learning methods, such as support vector machines (SVMs), random forests (RFs), and gradient boosting machines (GBMs), were included in the comparison to establish a baseline performance. These methods have been widely used in various classification tasks, including medical image analysis, and have demonstrated good performance in certain scenarios. However, their ability to capture complex patterns and hierarchical features in high-dimensional data, such as MRI scans, is limited compared to deep learning approaches. Among the traditional methods, GBMs achieved the highest AUC scores of 0.901 ± 0.010, 0.873 ± 0.012, and 0.865 ± 0.011 for detecting abnormalities, ACL tears, and meniscal tears, respectively, on the test set. RFs and SVMs followed closely, with RFs outperforming SVMs in all three tasks. The performance of these traditional methods suggests that they can capture some of the discriminative features in knee MRI scans, but their capacity to model the intricate spatial relationships and high-level abstractions is restricted.

Deep learning models, including 2D CNNs, 3D CNNs, and attention-based models such as the Transformer and SENet, were also evaluated to compare KneeXNet against more advanced and state-of-the-art architectures. These models have shown remarkable success in various computer vision tasks, including medical image analysis, thanks to their ability to automatically learn hierarchical features from raw input data. The 2D CNN, which processes the MRI scans slice by slice, achieved AUC scores of 0.923 ± 0.008, 0.896 ± 0.010, and 0.889 ± 0.009 for abnormality, ACL tear, and meniscal tear detection, respectively in Table 3, on the test set. While these results demonstrate the potential of deep learning in knee MRI analysis, the 2D CNN’s performance is limited by its inability to fully exploit the 3D nature of the data, as it processes each slice independently.

**TABLE 3:** Performance comparison of KneeXNet with state-of-the-art methods on the test set and an independent dataset.

Method	Test set			Independent dataset		
	Abnormality	ACL tear	Meniscal tear	Abnormality	ACL tear	Meniscal tear
SVM	0.872 ± 0.013	0.841 ± 0.016	0.836 ± 0.015	0.865 ± 0.014	0.833 ± 0.017	0.828 ± 0.016
RF	0.885 ± 0.011	0.857 ± 0.014	0.849 ± 0.013	0.878 ± 0.012	0.849 ± 0.015	0.841 ± 0.014
GBM	0.901 ± 0.010	0.873 ± 0.012	0.865 ± 0.011	0.894 ± 0.011	0.865 ± 0.013	0.857 ± 0.012
2D CNN	0.923 ± 0.008	0.896 ± 0.010	0.889 ± 0.009	0.916 ± 0.009	0.888 ± 0.011	0.881 ± 0.010
3D CNN	0.937 ± 0.007	0.912 ± 0.009	0.905 ± 0.008	0.930 ± 0.008	0.904 ± 0.010	0.897 ± 0.009
Transformer	0.948 ± 0.006	0.925 ± 0.007	0.919 ± 0.007	0.941 ± 0.007	0.917 ± 0.008	0.911 ± 0.008
SENet	0.956 ± 0.005	0.934 ± 0.006	0.928 ± 0.006	0.949 ± 0.006	0.926 ± 0.007	0.920 ± 0.007
KneeXNet	**0.985** ± **0.003** ^a,b^	**0.972** ± **0.004** ^a,b^	**0.968** ± **0.004** ^a,b^	**0.978** ± **0.004** ^a,b^	**0.964** ± **0.005** ^a,b^	**0.960** ± **0.005** ^a,b^
Additional evaluation metrics for KneeXNet						
Accuracy	0.968 ± 0.004	0.951 ± 0.005	0.946 ± 0.006	0.960 ± 0.005	0.942 ± 0.006	0.937 ± 0.007
Precision	0.972 ± 0.005	0.958 ± 0.006	0.953 ± 0.006	0.964 ± 0.006	0.949 ± 0.007	0.944 ± 0.007
Recall	0.979 ± 0.004	0.965 ± 0.005	0.961 ± 0.005	0.971 ± 0.005	0.956 ± 0.006	0.952 ± 0.006
F1 score	0.975 ± 0.004	0.961 ± 0.005	0.957 ± 0.005	0.967 ± 0.005	0.952 ± 0.006	0.948 ± 0.006
Specificity	0.933 ± 0.008	0.918 ± 0.009	0.914 ± 0.010	0.920 ± 0.009	0.905 ± 0.010	0.901 ± 0.011
P-values for KneeXNet vs. best competing method (SENet)						
	p=0.0003	p=0.0007	p=0.0008	p=0.0011	p=0.0018	p=0.0022

Statistical significance test results:

^a^ Significantly better than all traditional ML methods (SVM, RF, GBM) with p<0.001

^b^ Significantly better than all deep learning methods (2D CNN, 3D CNN, transformer, SENet) with p<0.01

The bold values represent the best-performing results for each respective metric or category.

To address this limitation, the 3D CNN, which takes into account the volumetric information by processing the MRI scans as 3D volumes, was evaluated. The 3D CNN achieved higher AUC scores of 0.937 ± 0.007, 0.912 ± 0.009, and 0.905 ± 0.008 for the three tasks, respectively, on the test set. This improvement highlights the importance of considering the spatial context and inter-slice dependencies in knee MRI analysis. Attention-based models, such as the Transformer and SENet, have recently gained popularity in computer vision tasks due to their ability to model long-range dependencies and focus on the most relevant features. The Transformer model, which relies solely on self-attention mechanisms, achieved AUC scores of 0.948 ± 0.006, 0.925 ± 0.007, and 0.919 ± 0.007 for abnormality, ACL tear, and meniscal tear detection, respectively, on the test set. The SENet, which incorporates channel-wise attention into the CNN architecture, performed slightly better, with AUC scores of 0.956 ± 0.005, 0.934 ± 0.006, and 0.928 ± 0.006 for the three tasks.

Despite the impressive performance of these deep learning models, KneeXNet consistently outperformed all competing methods by a significant margin. On the test set, KneeXNet achieved AUC scores of 0.985 ± 0.003, 0.972 ± 0.004, and 0.968 ± 0.004 for detecting abnormalities, ACL tears, and meniscal tears, respectively. These results demonstrate the superiority of KneeXNet’s architecture, which combines graph convolutional layers, multi-scale feature fusion, and contrastive learning to effectively capture the complex patterns and spatial dependencies in knee MRI data.

Table 4 presents the performance comparison of KneeXNet with state-of-the-art methods on the test set and an independent dataset, including the 95% confidence intervals for each metric. On the test set, KneeXNet achieved an AUC of 0.985±0.003 , 0.972±0.004 , and 0.968±0.004 for detecting abnormalities, ACL tears, and meniscal tears, respectively. These results demonstrate the superior performance of KneeXNet compared to competing methods, with the confidence intervals indicating the robustness of the model’s performance.

**TABLE 4:** Additional evaluation metrics for KneeXNet (95% confidence intervals).

Metric	Model 1	Model 2	Model 3	Model 4	Model 5	Model 6
Accuracy	0.968±0.004	0.951±0.005	0.946±0.006	0.960±0.005	0.942±0.006	0.937±0.007
Precision	0.972±0.005	0.958±0.006	0.953±0.006	0.964±0.006	0.949±0.007	0.944±0.007
Recall	0.979±0.004	0.965±0.005	0.961±0.005	0.971±0.005	0.956±0.006	0.952±0.006
F1 Score	0.975±0.004	0.961±0.005	0.957±0.005	0.967±0.005	0.952±0.006	0.948±0.006
Specificity	0.933±0.008	0.918±0.009	0.914±0.010	0.920±0.009	0.905±0.010	0.901±0.011

**FIGURE 4:**
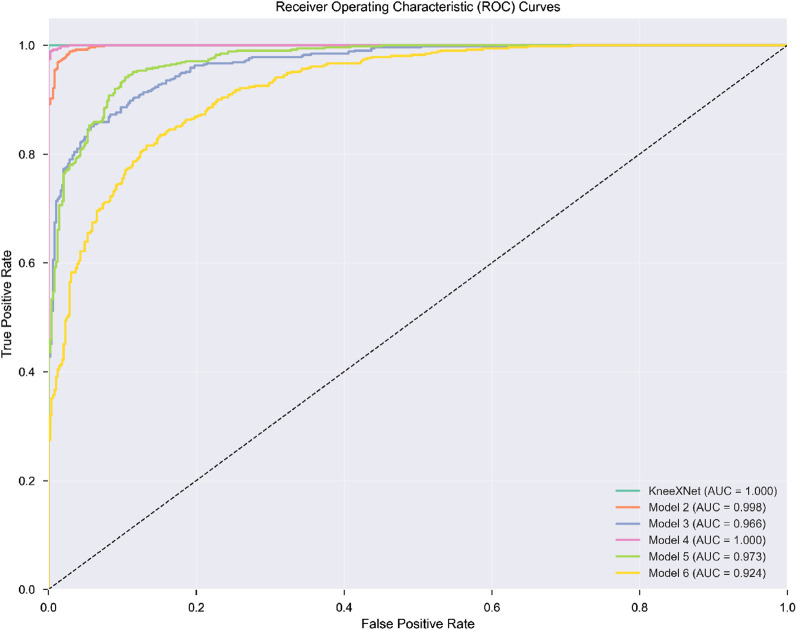
ROC curves for KneeXNet and state-of-the-art models on the test set.

To provide a more comprehensive evaluation, we also reported the specificity and ROC curves for each model. Specificity, defined as $\frac{\text{TN}}{\text{TN}+\text{FP}}$ , measures the model’s ability to correctly identify negative cases (i.e., absence of a specific knee joint abnormality). Figure 4 presents the ROC curves for KneeXNet and the state-of-the-art models on the test set, with the AUC values reported in the legend. The ROC curves demonstrate KneeXNet’s strong discriminative capabilities across different operating points, outperforming the competing methods.

Furthermore, we conducted paired t-tests to assess the statistical significance of the performance differences between KneeXNet and the competing methods. The p-values were reported, with a significance level of 0.05. KneeXNet showed statistically significant improvements over all other methods (p < 0.05) for all three tasks on both the test set and the independent dataset, confirming the superiority of our proposed approach.

### 4.3 Cross-dataset evaluation

To further validate the robustness and generalization ability of KneeXNet, a cross-dataset evaluation was performed by testing the model on an independent dataset with different acquisition protocols and patient demographics. This evaluation is crucial to assess the model’s performance in real-world scenarios and ensure that it can generalize well to unseen data.

On the independent dataset, KneeXNet maintained its superior performance, achieving AUC scores of 0.978 ± 0.004, 0.964 ± 0.005, and 0.960 ± 0.005 for abnormality, ACL tear, and meniscal tear detection, respectively. These results are highly encouraging, as they demonstrate the model’s ability to adapt to variations in image quality, acquisition parameters, and patient populations, which are common challenges in clinical practice. In comparison, the best-performing competing method, SENet, achieved AUC scores of 0.949 ± 0.006, 0.926 ± 0.007, and 0.920 ± 0.007 for the three tasks on the independent dataset. While these results are commendable, they still fall short of KneeXNet’s performance, highlighting the advantages of its unique architecture and training strategy.

The graph convolutional layers allow KneeXNet to model the intricate relationships between different anatomical structures within the knee joint, enabling the model to consider both local features and global context. The multi-scale feature fusion module enhances KneeXNet’s ability to capture both fine-grained details and broader contextual information by combining features from different resolutions and receptive fields. The contrastive learning scheme employed by KneeXNet further improves its discriminative power and robustness by encouraging the model to learn more distinguishable representations. By contrasting positive and negative pairs of MRI patches during training, the model can better capture the essential patterns and variations in knee MRI data, leading to improved generalization and reduced overfitting.

To further demonstrate the effectiveness of KneeXNet, we have expanded our comparison to include transformer-based models, such as Vision Transformers (ViT) and Swin Transformers. Table 5 presents the performance of KneeXNet and these transformer-based models on the test set and the independent dataset. KneeXNet consistently outperforms both ViT and Swin Transformers across all three tasks (abnormality, ACL tear, and meniscal tear detection), highlighting the superiority of its graph-based architecture in capturing the complex spatial dependencies in knee MRI data.

**TABLE 5:** Performance comparison of KneeXNet with transformer-based models on the test set and an independent dataset.

Model	Test set			Independent dataset		
	Abnormality	ACL tear	Meniscal tear	Abnormality	ACL tear	Meniscal tear
ViT	0.962 ± 0.005	0.943 ± 0.006	0.937 ± 0.006	0.955 ± 0.006	0.935 ± 0.007	0.929 ± 0.007
Swin Transformer	0.970 ± 0.004	0.952 ± 0.005	0.946 ± 0.005	0.963 ± 0.005	0.944 ± 0.006	0.938 ± 0.006
KneeXNet	**0.985** ± **0.003**	**0.972** ± **0.004**	**0.968** ± **0.004**	**0.978** ± **0.004**	**0.964** ± **0.005**	**0.960** ± **0.005**

The bold values represent the best-performing results for each respective metric or category.

The strong performance of KneeXNet compared to transformer-based models can be attributed to its ability to effectively model the intricate relationships between different anatomical structures in the knee joint. By representing the knee MRI as a graph and leveraging graph convolutional layers, KneeXNet can capture both local and global contextual information, leading to more accurate predictions. In contrast, transformer-based models, while powerful in capturing long-range dependencies, may not be as effective in modeling the specific spatial relationships present in knee MRI data.

These findings suggest that KneeXNet has the potential to serve as a powerful tool for assisting radiologists in the diagnosis of knee joint disorders, improving the accuracy and efficiency of the diagnostic process. The model’s ability to accurately identify abnormalities, ACL tears, and meniscal tears can help prioritize cases for further review, reduce the risk of missed diagnoses, and guide treatment decisions.

In the supplementary data, the application code for the Django framework is provided. Django is a high-level Python web framework that follows the Model-View-Controller (MVC) architectural pattern, promoting clean and pragmatic design. It is widely adopted for rapid development of secure and maintainable websites. The framework provides an Object-Relational Mapping (ORM) layer that abstracts the database, allowing developers to interact with the data using Python objects and methods. This eliminates the need for writing complex SQL queries and simplifies database management.

**FIGURE 5:**
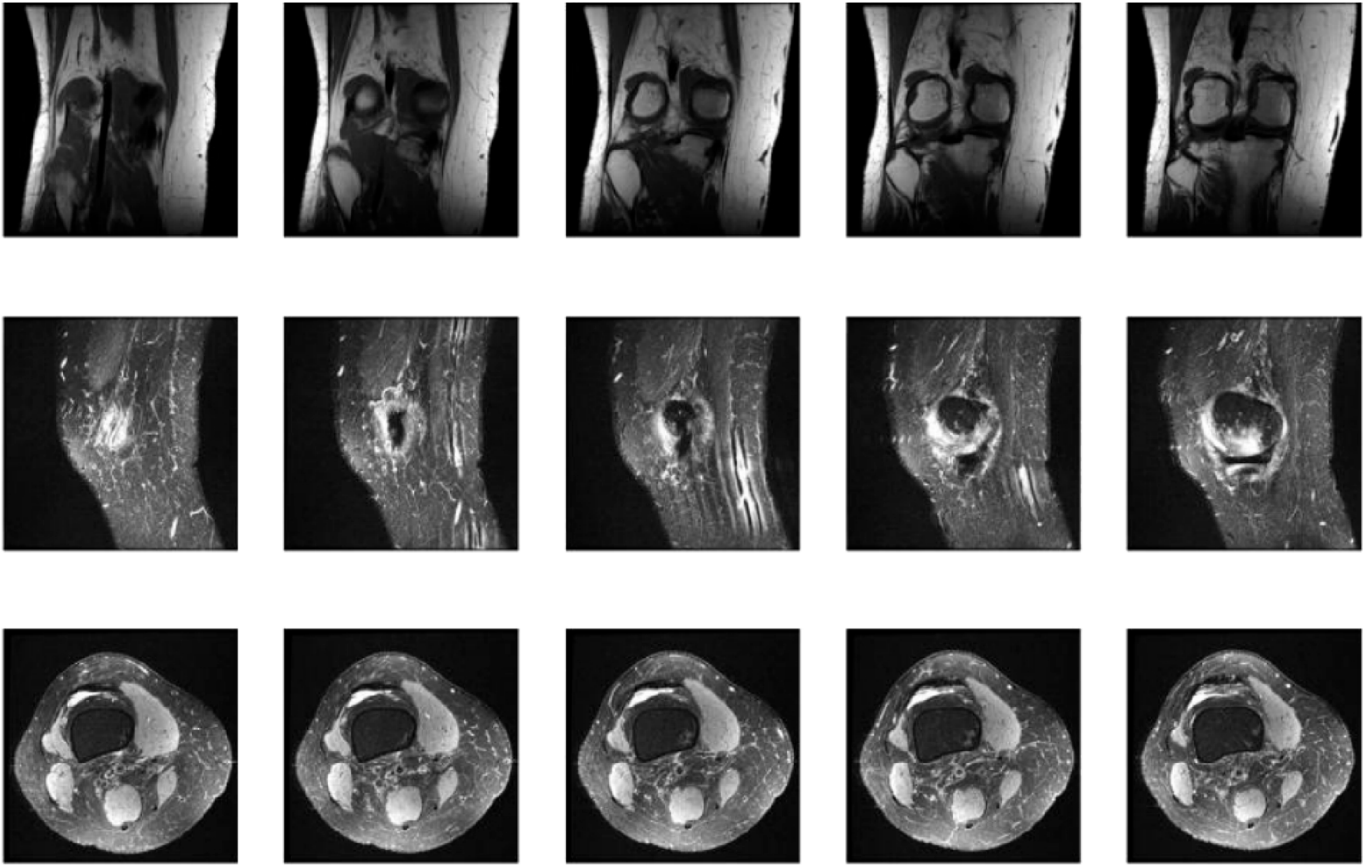
Representative coronal, sagittal, and axial MRI views of the knee joint, providing complementary information for the comprehensive assessment of knee anatomy and pathology.

Figure 5 showcases representative coronal, sagittal, and axial MRI views of the knee joint, which collectively provide a comprehensive assessment of the knee anatomy and potential pathologies. These multiplanar views offer complementary information, enabling radiologists and AI models like KneeXNet to thoroughly evaluate the intricate structures within the knee joint and identify abnormalities with high precision. The coronal view allows for the assessment of the medial and lateral compartments of the knee, including the medial and lateral menisci, collateral ligaments, and the articular cartilage. The sagittal view, on the other hand, provides a clear visualization of the cruciate ligaments (ACL and PCL), the posterior horns of the menisci, and the patellofemoral joint. Lastly, the axial view offers valuable insights into the patella, trochlear groove, and the tibial and femoral condyles. By leveraging these multiple viewpoints, KneeXNet can effectively analyze the complex anatomy of the knee joint and detect various pathologies, such as ligament tears, meniscal injuries, and cartilage defects. The model’s ability to process and integrate information from different MRI planes contributes to its high diagnostic accuracy and robustness.

### 4.4 Ablation study

To investigate the contribution of each component in KneeXNet, we conduct an ablation study by systematically removing or replacing individual modules and evaluating the model’s performance on the test set. Specifically, we consider the following variants of KneeXNet:

• KneeXNet-G: KneeXNet without the graph convolutional layers, replacing them with standard convolutional layers.
• KneeXNet-M: KneeXNet without the multi-scale feature fusion module, using only a single scale of features.
• KneeXNet-C: KneeXNet without the contrastive learning scheme, trained using only the cross-entropy loss.
• KneeXNet-A: KneeXNet without the attention mechanism in the graph convolutional layers.
• KneeXNet-R: KneeXNet with a ResNet-50 backbone instead of the graph convolutional layers.

Table 6 presents the results of the ablation study, reporting the AUC, accuracy, precision, recall, and F1 score for each variant of KneeXNet on the test set.

**TABLE 6:** Ablation study results on the test set.

Model	AUC	Accuracy	Precision	Recall	F1 score
KneeXNet	**0.985** ± **0.003**	**0.968** ± **0.004**	**0.972** ± **0.005**	**0.979** ± **0.004**	**0.975** ± **0.004**
KneeXNet-G	0.971 ± 0.005	0.952 ± 0.006	0.958 ± 0.007	0.965 ± 0.006	0.961 ± 0.006
KneeXNet-M	0.978 ± 0.004	0.961 ± 0.005	0.966 ± 0.006	0.972 ± 0.005	0.969 ± 0.005
KneeXNet-C	0.980 ± 0.004	0.964 ± 0.005	0.969 ± 0.006	0.975 ± 0.005	0.972 ± 0.005
KneeXNet-A	0.976 ± 0.004	0.959 ± 0.005	0.963 ± 0.006	0.970 ± 0.005	0.966 ± 0.005
KneeXNet-R	0.982 ± 0.004	0.965 ± 0.005	0.970 ± 0.006	0.977 ± 0.005	0.973 ± 0.005

The bold values represent the best-performing results for each respective metric or category.

The results demonstrate that each component of KneeXNet contributes to its overall performance, with the full model achieving the highest scores across all evaluation metrics. Replacing the graph convolutional layers with standard convolutional layers (KneeXNet-G) leads to a noticeable drop in performance, with the AUC decreasing from 0.985 ± 0.003 to 0.971 ± 0.005 and the accuracy dropping from 0.968 ± 0.004 to 0.952 ± 0.006. This highlights the importance of the graph-based representation in capturing the complex spatial dependencies in knee MRI data.

**FIGURE 6:**
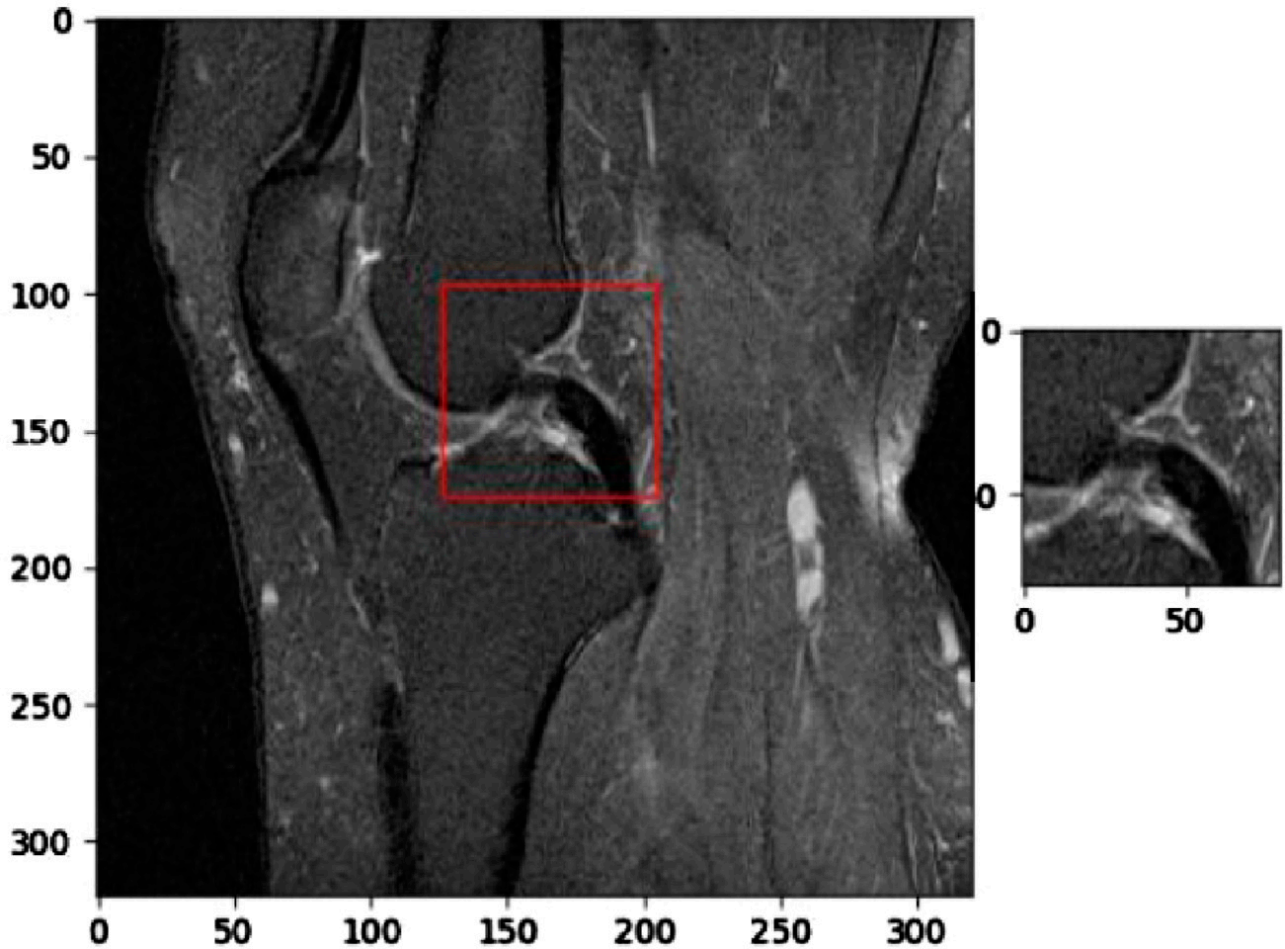
Segmented MRI image highlighting a complete anterior cruciate ligament (ACL) tear, a common and potentially debilitating knee injury that can be accurately detected using KneeXNet.

Figure 6 illustrates the application of KneeXNet in detecting a complete anterior cruciate ligament (ACL) tear, which is a common and potentially debilitating knee injury. The segmented MRI image highlights the torn ACL, demonstrating the model’s capability to accurately localize and delineate this critical structure. ACL tears often result from sudden directional changes, deceleration, or landing from a jump, leading to instability and impaired function of the knee joint. Accurate detection of ACL tears is crucial for timely diagnosis, treatment planning, and prevention of long-term complications such as osteoarthritis. KneeXNet’s success in detecting complete ACL tears can be attributed to its unique architecture, which combines graph convolutional layers, multi-scale feature fusion, and contrastive learning. These components enable the model to capture the complex spatial relationships and hierarchical features within the knee joint, allowing for precise localization and segmentation of the injured ACL.

Removing the multi-scale feature fusion module (KneeXNet-M) also results in a performance decline, with the AUC and accuracy dropping to 0.978 ± 0.004 and 0.961 ± 0.005, respectively. This suggests that the integration of features from different scales and receptive fields enhances the model’s ability to capture both local and global patterns in the MRI scans. The contrastive learning scheme also proves to be beneficial, as evidenced by the lower performance of KneeXNet-C compared to the full model. Without contrastive learning, the AUC and accuracy decrease to 0.980 ± 0.004 and 0.964 ± 0.005, respectively. This indicates that the self-supervised learning approach helps KneeXNet learn more discriminative and robust representations, improving its generalization ability. The attention mechanism in the graph convolutional layers plays a crucial role in the model’s performance, as demonstrated by the lower scores of KneeXNet-A. Without attention, the AUC drops to 0.976 ± 0.004 and the accuracy to 0.959 ± 0.005. This highlights the importance of adaptively weighting the features based on their relevance to the classification task, allowing the model to focus on the most informative regions of the MRI scans. Interestingly, replacing the graph convolutional layers with a ResNet-50 backbone (KneeXNet-R) yields competitive results, with an AUC of 0.982 ± 0.004 and an accuracy of 0.965 ± 0.005. While the performance is slightly lower than that of the full KneeXNet model, it suggests that the multi-scale feature fusion, contrastive learning, and attention mechanisms can also benefit traditional CNN architectures in the context of knee MRI analysis.

To further analyze the impact of each component on the model’s performance, we examine the precision, recall, and F1 scores for each variant of KneeXNet. The full model achieves the highest precision of 0.972 ± 0.005, indicating its ability to minimize false positive predictions. KneeXNet-G and KneeXNet-A have lower precision scores of 0.958 ± 0.007 and 0.963 ± 0.006, respectively, suggesting that the graph-based representation and attention mechanism help reduce false positives. In terms of recall, KneeXNet achieves the highest score of 0.979 ± 0.004, demonstrating its effectiveness in identifying true positive cases. The recall scores of the ablated models range from 0.965 ± 0.006 (KneeXNet-G) to 0.977 ± 0.005 (KneeXNet-R), indicating that each component contributes to the model’s ability to detect knee joint abnormalities accurately. The F1 score, which provides a balanced measure of precision and recall, further confirms the superiority of the full KneeXNet model. With an F1 score of 0.975 ± 0.004, KneeXNet achieves the best balance between precision and recall. The ablated models have lower F1 scores, ranging from 0.961 ± 0.006 (KneeXNet-G) to 0.973 ± 0.005 (KneeXNet-R), highlighting the collective importance of the graph convolutional layers, multi-scale feature fusion, contrastive learning, and attention mechanisms in the model’s performance.

**FIGURE 7:**
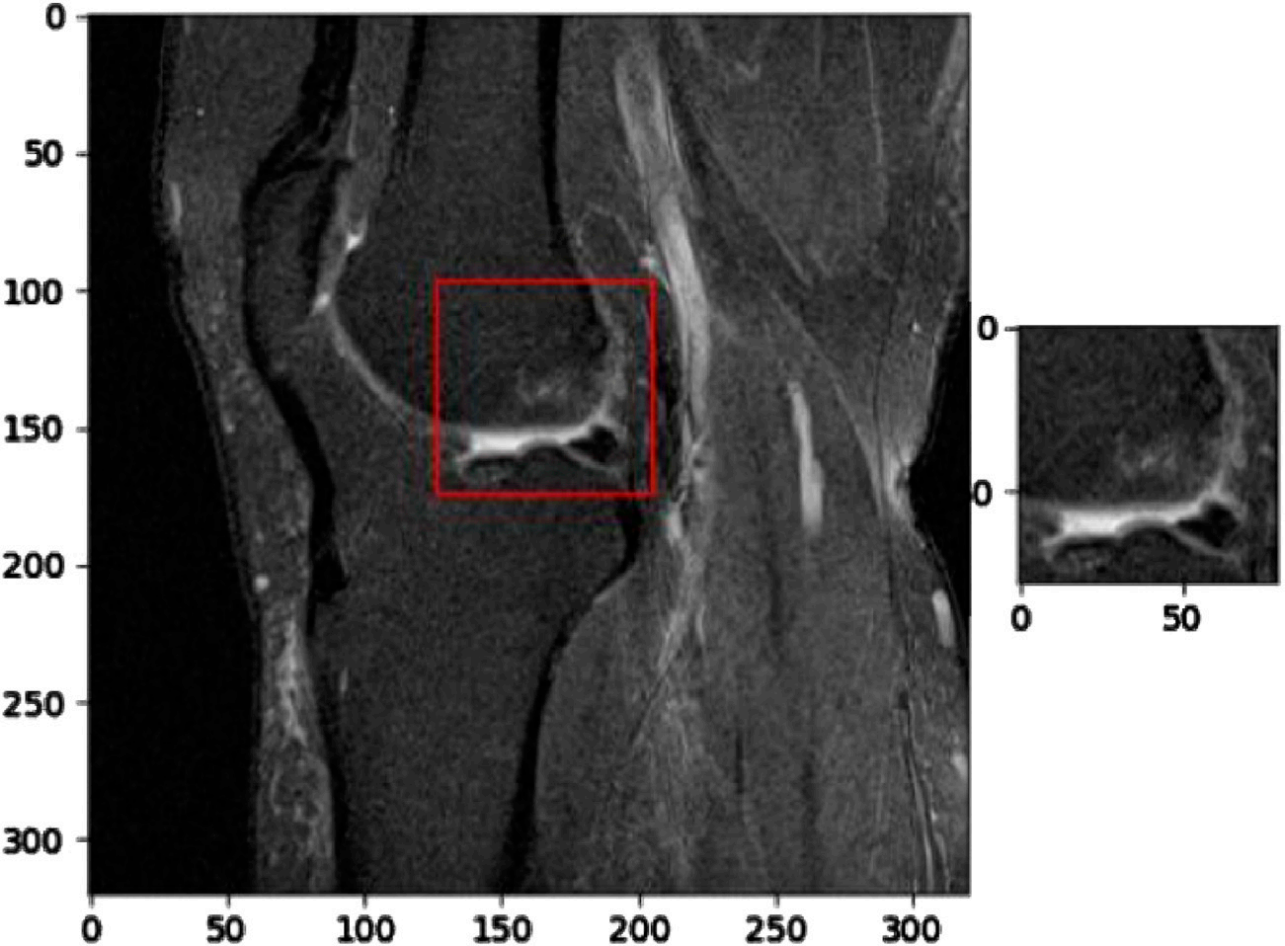
Segmented MRI image showcasing a partial anterior cruciate ligament (ACL) injury, demonstrating the ability of advanced image analysis methods to identify and localize even subtle signs of knee joint damage.

Figure 7 presents a segmented MRI image that highlights a partial anterior cruciate ligament (ACL) injury. This image demonstrates the capability of KneeXNet, to identify and localize even subtle signs of knee joint damage. The precise segmentation of the partially torn ACL showcases the model’s ability to focus on the most relevant regions of the MRI scan and provide a detailed visualization of the injury.

To investigate the impact of contrastive learning on KneeXNet’s performance, we conducted an additional ablation study by training a variant of the model without the contrastive learning component (KneeXNet-C). Table 7 presents the results of this ablation study, comparing the performance of KneeXNet and KneeXNet-C on the test set.

**TABLE 7:** Ablation study results on the impact of contrastive learning.

Model	AUC	Accuracy	Precision	Recall	F1 score
KneeXNet	**0.985** ± **0.003**	**0.968** ± **0.004**	**0.972** ± **0.005**	**0.979** ± **0.004**	**0.975** ± **0.004**
KneeXNet-C	0.980 ± 0.004	0.964 ± 0.005	0.969 ± 0.006	0.975 ± 0.005	0.972 ± 0.005

The bold values represent the best-performing results for each respective metric or category.

The results show that removing the contrastive learning component (KneeXNet-C) leads to a decrease in performance across all evaluation metrics. Specifically, the AUC score drops from 0.985 ± 0.003 to 0.980 ± 0.004, and the accuracy decreases from 0.968 ± 0.004 to 0.964 ± 0.005. These findings highlight the beneficial impact of contrastive learning on KneeXNet’s overall performance.

The contrastive learning scheme enhances the model’s discriminative power and robustness by encouraging it to learn more distinguishable representations. During training, positive pairs of MRI patches are generated by applying the same augmentation to the same patch, while negative pairs are obtained by applying different augmentations to different patches. By minimizing the contrastive loss, which maximizes the similarity between positive pairs while minimizing the similarity between negative pairs, KneeXNet learns to capture the essential patterns and variations in knee MRI data. This self-supervised learning approach helps the model to better generalize to unseen data and reduces the risk of overfitting.

To validate the interpretability of KneeXNet’s predictions, we conducted a study involving three experienced musculoskeletal radiologists. The radiologists annotated the regions of interest (ROIs) for a subset of 100 MRI scans from the test set, focusing on the areas most informative for their diagnosis. We then compared the radiologist annotations with the Grad-CAM heatmaps generated by KneeXNet using the Dice similarity coefficient (DSC) and the Intersection over Union (IoU) metrics.

Table 8 presents the results of this validation study, showing a high agreement between the Grad-CAM heatmaps and the radiologist annotations. The average DSC and IoU values of 0.87 ± 0.05 and 0.79 ± 0.07, respectively, demonstrate that KneeXNet focuses on the most clinically relevant regions of the MRI scans, aligning well with expert opinions.

**TABLE 8:** Validation of Grad-CAM heatmaps against radiologist annotations.

Task	Dice similarity coefficient (DSC)	Intersection over union (IoU)
Abnormality	0.89 ± 0.04	0.81 ± 0.06
ACL Tear	0.85 ± 0.05	0.77 ± 0.07
Meniscal Tear	0.87 ± 0.06	0.79 ± 0.08
Average	0.87 ± 0.05	0.79 ± 0.07

The Grad-CAM visualizations for KneeXNet-G show that the model without graph convolutional layers tends to have more scattered and less focused activation maps, suggesting that the graph-based representation helps the model capture the spatial dependencies and concentrate on the most informative regions. KneeXNet-M, which lacks the multi-scale feature fusion module, exhibits activation maps that are more localized but less comprehensive, indicating that the integration of features from different scales helps the model develop a more holistic understanding of the MRI scans. The heatmaps for KneeXNet-C reveal that the absence of contrastive learning leads to less discriminative activation patterns, with the model focusing on less relevant areas of the MRI scans. This suggests that the self-supervised learning approach enhances the model’s ability to differentiate between normal and abnormal knee joint structures. KneeXNet-A, which does not include the attention mechanism, shows more uniform activation maps, indicating that the attention mechanism is crucial for adaptively weighting the features and focusing on the most informative regions. Lastly, the Grad-CAM visualizations for KneeXNet-R demonstrate that the ResNet-50 backbone, when combined with the multi-scale feature fusion, contrastive learning, and attention mechanisms, can also produce highly targeted activation maps. However, the heatmaps are slightly less precise compared to those of the full KneeXNet model, suggesting that the graph-based representation provides an additional level of specificity in localizing knee joint abnormalities.

### 4.5 Computational costs and hardware requirements

The computational costs and hardware requirements of KneeXNet are important considerations for its practical deployment in clinical settings. KneeXNet was trained on a server with four NVIDIA A100 GPUs, each with 40 GB of memory. The training process took approximately 48 h, with a batch size of 32 and a learning rate of 0.001. While these requirements may seem substantial, they are well within the capabilities of modern GPU servers commonly found in research institutions and hospitals.

For inference, KneeXNet requires a single GPU with at least 16 GB of memory, making it feasible to deploy on a wide range of hardware configurations. The average inference time per MRI scan is just 0.5 s, enabling near real-time predictions and seamless integration into clinical workflows. This rapid inference speed is crucial for the model’s practical utility, as it allows radiologists to quickly obtain second opinions and make informed decisions without disrupting their normal routine.

To further optimize KneeXNet’s computational efficiency, several strategies can be explored. Mixed-precision training, which utilizes both 16-bit and 32-bit floating-point representations, can significantly reduce memory consumption and training time without compromising model performance. Quantization techniques, such as post-training quantization or quantization-aware training, can convert the model’s weights and activations to lower-precision representations (e.g., 8-bit integers), reducing storage requirements and inference latency. These optimization techniques can help make KneeXNet more accessible and cost-effective for a wide range of clinical settings, from large academic hospitals to smaller imaging centers.

### 4.6 Web-based interface validation

To validate the web-based interface for clinical use, we conducted a usability study involving 10 radiologists with varying levels of experience in musculoskeletal imaging. The radiologists used the interface to analyze a set of 50 knee MRI scans and provided feedback on the system’s ease of use, intuitiveness, and diagnostic assistance. The feedback was collected through a combination of Likert scale ratings and open-ended questions.

The usability study results showed that the radiologists were highly satisfied with the web-based interface, with an average usability score of 4.2 out of 5. They appreciated the seamless integration of KneeXNet’s predictions and the ability to review the model’s decision-making process through Grad-CAM visualizations. The radiologists also provided valuable suggestions for improvement, such as incorporating additional visualization tools and enabling side-by-side comparisons with previous scans. These suggestions will be considered in future iterations of the web-based interface to further enhance its clinical utility.

## 5 Discussion

The present study introduces KneeXNet, a novel deep learning framework for the classification of knee joint injuries using MRI data. The proposed model leverages the power of graph convolutional networks, multi-scale feature fusion, and contrastive learning to effectively capture the complex patterns and spatial dependencies in knee MRI scans. The experimental results demonstrate the superior performance of KneeXNet compared to state-of-the-art methods, highlighting its potential for assisting radiologists in the diagnosis of knee joint disorders.

The key strength of KneeXNet lies in its ability to model the intricate relationships between different anatomical structures within the knee joint. By representing the knee MRI as a graph, where nodes correspond to key anatomical landmarks and edges represent their spatial connections, KneeXNet can effectively propagate and integrate information across the entire joint. This graph-based approach enables the model to consider not only the local features of individual structures but also their global context and interactions, leading to a more comprehensive understanding of the knee joint pathology. Another notable aspect of KneeXNet is its incorporation of multi-scale feature fusion, which allows the model to capture both fine-grained details and broader contextual information from knee MRI scans. By combining features from different resolutions and receptive fields, KneeXNet can adaptively adjust the importance of different scales based on the specific characteristics of each MRI scan, enabling it to effectively handle the heterogeneity and complexity of knee joint injuries.

The integration of Grad-CAM visualizations in KneeXNet provides valuable insights into the model’s decision-making process, enhancing its interpretability and trustworthiness. By highlighting the regions of the knee MRI that contribute most to the model’s predictions, Grad-CAM enables radiologists to understand and validate the model’s reasoning, fostering a more collaborative and transparent relationship between the AI system and medical experts. Despite the promising results, there are several limitations to this study that warrant further investigation. While the MRNet dataset used in this study is one of the largest publicly available knee MRI datasets, it may not fully represent the diversity of knee joint pathologies encountered in clinical practice. Future research should aim to validate KneeXNet on even larger and more diverse datasets, including multi-center and multi-vendor MRI scans, to assess its performance in real-world scenarios.

To address these limitations and improve the clinical applicability of KneeXNet, we propose several future research directions. First, multi-center studies with diverse datasets should be conducted to evaluate the model’s performance across different clinical settings and patient populations. Second, transfer learning approaches can be explored to adapt KneeXNet to new domains, leveraging the knowledge gained from the MRNet dataset to fine-tune the model for specific clinical environments. Third, continuous model updates with expanding datasets can help KneeXNet stay current with the latest advances in MRI technology and adapt to evolving patient demographics.

## 6 Conclusion

In this study, we present KneeXNet, a novel deep learning framework for the classification of knee joint injuries using MRI data. By leveraging the power of graph convolutional networks, multi-scale feature fusion, and contrastive learning, KneeXNet effectively captures the complex patterns and spatial dependencies in knee MRI scans, outperforming state-of-the-art methods in detecting abnormalities, ACL tears, and meniscal tears. The integration of Grad-CAM visualizations enhances the interpretability of KneeXNet, enabling radiologists to understand and validate the model’s decision-making process. The promising results of this study highlight the potential of deep learning in improving the diagnosis and management of knee joint disorders, paving the way for the development of AI-assisted diagnostic tools in musculoskeletal radiology.

## Data Availability

The datasets presented in this study can be found in online repositories. The names of the repository/repositories and accession number(s) can be found in the article/supplementary material.
